# Risk Factors for Coronary Events After Robotic Hybrid Off-Pump Coronary Revascularization

**DOI:** 10.3390/jcdd12010021

**Published:** 2025-01-10

**Authors:** Aleksander Dokollari, Beatrice Bacchi, Serge Sicouri, Francesco Cabrucci, Massimo Bonacchi, Danielle Spragan, Mary Ann C. Wertan, Nitin Ghorpade, Stephanie Kjelstrom, Georgia Montone, Yoshiyuki Yamashita, Basel Ramlawi, Francis Sutter

**Affiliations:** 1 Department of Cardiac Surgery Research, Lankenau Institute for Medical Research, Main Line Health, Wynnewood, PA 19096, USA; sicouris@mlhs.org (S.S.); francesco.cabrucci@unifi.it (F.C.); spragand@mlhs.org (D.S.); kjelstroms@mlhs.org (S.K.); montoneg@mlhs.org (G.M.); yamashitay@mhls.org (Y.Y.); ramlawib@mlhs.org (B.R.); 2 Cardiac Surgery Division, St. Boniface Hospital, University of Manitoba, Winnipeg, MB R3T 2N2, Canada; nitin.ghorpade@vch.ca; 3 F.U. of Cardiac Surgery, Clinical and Experimental Medicine Department, University of Firenze, 50121 Florence, Italy; beatrice.bacchi@unifi.it (B.B.); massimo.bonacchi@unifi.it (M.B.); 4 Department of Cardiac Surgery, Lankenau Heart Institute, Main Line Health, Wynnewood, PA 19096, USA; wertanm@mlhs.org (M.A.C.W.); sutterf@mhls.org (F.S.)

**Keywords:** coronary artery bypass graft, myocardial revascularization, robotic cardiac surgery, percutaneous coronary revascularization

## Abstract

Objectives: The impact of long-term complications after robotic hybrid coronary revascularization (HCR), including persistent angina, repeat revascularization, and myocardial infarction (MI), remains limited. This study aims to determine the risk factors for coronary events after robotic HCR and their time-varying effects on outcomes. Methods: We identified all consecutive patients who underwent robotic HCR at our institution. Baseline characteristics were explored as possible risk factors for angina, MI, and repeat revascularization with stents at any time during the follow-up. Results: A total of 875 patients (mean age 71.1 ± 11.1 years) were included. After a median follow-up of 3.32 years (IQR 1.18–6.34 years), angina occurred in 134 patients (15.3%), repeat revascularization with stents in 139 patients (15.8%), and MI in 36 patients (4.1%). The hazard rates for all outcomes increased with follow-up time, with a notable early rise around two years of follow-up for angina and, to a lesser extent, repeat revascularization. The risk factors were the lack of radial artery graft use, black race, diabetes, obesity, chronic obstructive pulmonary disease, low ejection fraction <50%, severe left main coronary artery stenosis (>50%), and more than three-vessel disease. Conclusions: Optimization of modifiable periprocedural risk factors may positively impact long-term prognosis in patients undergoing robotic HCR.

## 1. Introduction

Clinical long-term complications after coronary artery bypass grafting (CABG) include persistent angina, myocardial infarction (MI), and repeat revascularization. Contemporary trials have assessed the long-term complication rate for both CABG and percutaneous coronary intervention (PCI) revascularization [1,2]. The NOBLE trial [1], comparing PCI versus CABG outcomes, reported an incidence of 6% vs. 2% for MI, 15% vs. 10% for repeat revascularization, and 5% vs. 2% for stroke, respectively. Five-year results from the EXCEL trial [2] reported an incidence of 10.6% vs. 9.1% of MI, 3.3% vs. 5.2% of stroke, and 16.9% vs. 10% of repeat revascularization for PCI vs. CABG, respectively. Clinical studies have also found several risk factors that impact long-term outcomes, such as insulin-dependent diabetes [3], age, gender, and race [4], among others. However, most of these retrospective studies included non-propensity matched patients [3,4], and no study has analyzed the impact of risk factors on patients undergoing hybrid coronary revascularization (HCR) [5]. We sought to determine the impact of preoperative risk factors associated with long-term prognosis in patients undergoing off-pump hybrid minimally invasive direct coronary artery bypass (MIDCAB) HCR.

## 2. Materials and Methods

### 2.1. Study Population

We identified all consecutive patients who underwent robotic off-pump HCR between February 2006 and June 2021 at our institution. The local Institutional Review Board (IRB 45CFR164.512) approved this study. As per institutional policy, patients undergoing HCR had their PCI within 1 week of the surgical procedure. In addition, patients undergoing HCR and PCI had ASA and clopidogrel for 1 year.

### 2.2. Data Collection

Patients’ characteristics were collected prospectively in a dedicated institutional registry. For our clinical study, inclusion criteria included patients aged ≥18 years and ≤90 years, as well as patients undergoing CABG with off-pump MIDCAB HCR. Decision on robotic versus standard CABG was made based on heart-team evaluation (Supplemental Table S1). Patients who were not amenable to traditional CABG were selected for MIDCAB HCR. LAD is bypassed with a LITA-LAD anastomosis through a 4 cm left minithoracotomy, and the other vessels are treated with PCI within 7 days of the surgical procedure. Dual antiplatelet therapy began on postoperative day 3 and was continued for 1 year after the procedure.

The outcomes of interest were the following: all-cause death; MACCE; stroke; angina; myocardial infarction (MI); and repeat revascularization at any time during the follow-up.

Patients were identified in a digital operation registry cardiac surgery database for all CABG operations, and data were collected retrospectively from charts. Follow-up was conducted at our outpatient clinic and from the hospital registry. In case the patient was not present at the appointment, we contacted the referring cardiologist to acquire the necessary information for this study. During the study period, eleven surgeons performed CABG procedures in our institution. Robotic-assisted MIDCAB procedure, HCR, and radial artery graft use are described in Supplemental Document S2.

### 2.3. Statistical Analysis

Descriptive statistics were used for preoperative, intraoperative, and postoperative characteristics. Continuous variables were assessed for normality and presented as means (standard deviation) or medians (interquartile range). We calculated the creatinine clearance value with the Cockcroft–Gault equation. All definitions were according to the STS definitions (Supplemental Document S1). To depict the effect of HCR on long-term prognosis, Kaplan–Meier cumulative survival curves were constructed for all-cause death, MACCE (death + MI + Stroke + repeat revascularization), MI, stroke, angina, and repeat revascularization. To find risk factors for our long-term outcomes, multivariable Cox and Fines and Gray regression models were built by first using a stepwise selection process with a higher *p*-value (0.5). Variables that met these criteria were entered into another stepwise selection in the order determined by the first. These nested models were tested with the Akaike information criterion (AIC), with the lowest AIC determining the final model. As these models are for hypothesis-generating purposes, no cross-validation or bootstrapping was conducted [6]. To calculate HR for all-cause mortality, we used Cox proportional HR and Fine and Grays sub-hazard ratios to account for competing risks for MI, stroke, repeat revascularization, and angina. We further evaluated the risk factors for each long-term outcome using the same statistics and in separate models at exit times of two, four, and eight years [6,7]. A *p*-value < 0.05 was considered significant, while 95% confidence intervals were reported. All analyses were performed using Stata 17.0 (Statacorp, LLC, College Station, TX, USA).

### 2.4. Patients’ Selection Process

Patients that may have been suitable candidates for robotic-assisted CABG had either isolated proximal left anterior descending (LAD) artery disease or multivessel disease in which the non-LAD artery disease was amenable to PCI with DES. On the other hand, patients with multivessel disease referred for HCR strategy were discussed as part of a heart-team approach with general and interventional cardiology and cardiac surgery. Indications for robotic-assisted CABG included (1) chronic total obstruction (CTO) of the LAD which can be associated with a second coronary artery disease lesion suitable for PCI/stent; (2) proximal LAD disease in patients in which a stent for LAD is considered not ideal due to young age or the length of stent to be deployed would increase the risk of in-stent restenosis; (3) patients with severe CAD and advanced age or comorbidities. The presence of multiple comorbidities combined with other factors increases the risk of perioperative complications and possibly mortality (STS PROM 3–10%); (4) patients with isolated left main (LM) coronary artery disease (CAD) and no (or minimal) artery disease in the other sites; include patients in which a conventional sternotomy CABG would result in CABG with two grafts and in which LAD would receive a left internal thoracic artery (LITA) anastomosis and the circumflex artery would not be bypassed with a second arterial conduit. LITA-to-LAD bypass is feasible and recommended by the heart team, but complete surgical revascularization with sternotomy and cardiopulmonary bypass (CPB) may be at high risk. The comorbidities may include any or in combination but are not limited to cerebrovascular disease, peripheral vascular disease (PVD osteoporosis), chronic kidney disease (CKD), poorly controlled diabetes, advanced age, autoimmune disorders, recent orthopedic disorder, chronic anemia; (5) patients with proximal LAD disease and CKD when LITA-to-LAD bypass is strongly recommended by the heart team and feasible, creatinine levels are chronically elevated but stable; there is no evidence of acute renal insufficiency; the STS PROM is moderate (3–8%); patients are not suitable for either complete revascularization by PCI or PCI to the LAD or LM coronary arteries; (6) patients awaiting transcatheter valve intervention, when patients were referred and accepted for transcatheter valve intervention but were found to have significant coronary artery disease. LITA-to-LAD bypass is recommended and feasible, but combined valve/CABG is at very elevated risk (STS PROM > 8%); (7) special circumstances in which patients decline CABG with sternotomy despite an appropriate conversation with surgeon and interventional cardiologist of the benefits of CABG over PCI.

In our early experience, patients with low STS-PROM risk scores, favorable body habitus, and good sizeable coronary targets were considered for robotic-assisted CABG. As experience was gained, patients with high-risk scores, independently of body habits, as well as with calcified coronary arteries, were added as robotic-assisted CABG candidates.

## 3. Results

Between February 2006 and June 2021, 875 patients (mean age 71.1 ± 11.1 years) underwent robotic HCR at our institution. Baseline demographics and procedural characteristics are summarized in Table 1.

Postoperative characteristics evidenced are summarized in Table 2.

**Figure 1:**
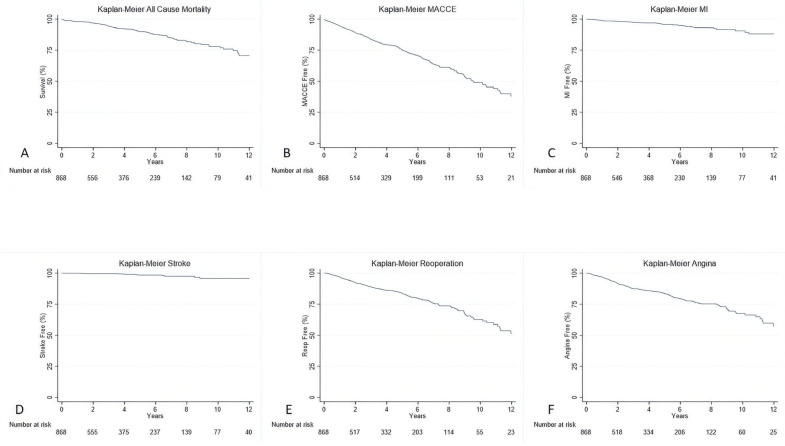
Kaplan–Meier curves with all-cause death (**A**), MACCE (**B**), MI (**C**), Stroke (**D**), Repeat Revascularization (**E**), and Angina (**F**).

All patients had at least one follow-up time point available. After a median follow-up of 3.32 years (IQR 1.18–6.34 years), angina occurred in 134 patients (15.3%), repeat revascularization with stents in 139 patients (15.8%), and MI in 36 patients (4.1%). In addition, all-cause death occurred in 87 (9.9%) patients, while MACCE occurred in 278 (31.8%) patients. Rates of all-cause death, MACCE, stroke, angina, repeat revascularization, and MI are shown in Figure 1.

At 15-year follow-up, overall risk factors associated with all-cause death and MACCE included age > 65 years, peripheral vascular disease (PVD), diabetes, BMI > 30 kg/m^2^, EF < 50%, dialysis, chronic obstructive pulmonary disease (COPD), and >three diseased vessels (Table 3). Risk factors associated with MI included lack of radial artery graft, while risk for stroke included black race (Table 3).

Risk factors associated with repeat revascularization and angina included BMI, >three diseased vessels, severe coronary vessel lesions (>70%), and severe left main coronary artery stenosis (>50%). Upon follow-up, HR for all outcomes increased, especially with an early rise around 2 years of follow-up for angina and repeat revascularization.

### Time-Varying Hazard Ratios

**Figure 2:**
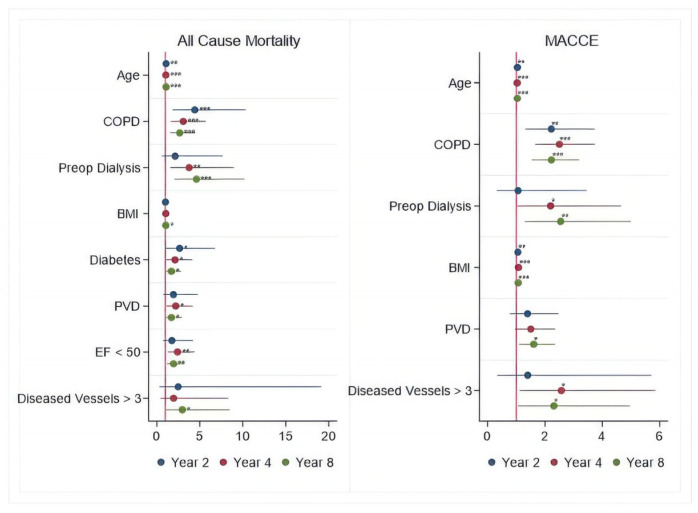
Periprocedural Risk Predictors for All-cause Death and MACCE. *—risk factor; **—important risk factor; ***—extremely important risk factor.

**Figure 3:**
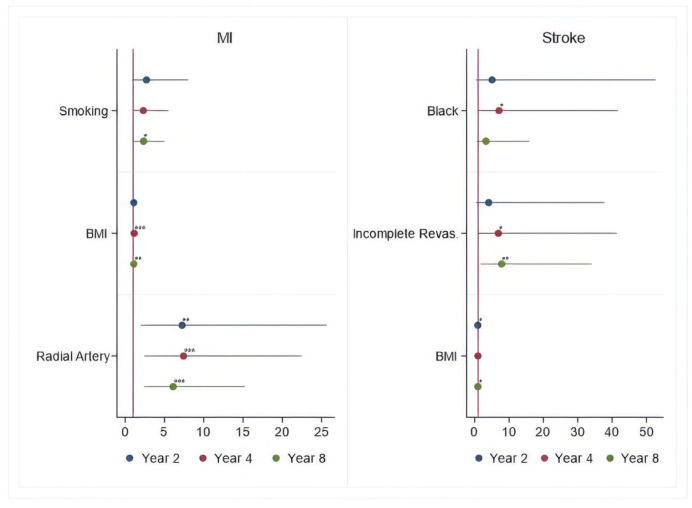
Periprocedural Risk Predictors for MI and Stroke. *—risk factor; **—important risk factor; ***—extremely important risk factor.

**Figure 4:**
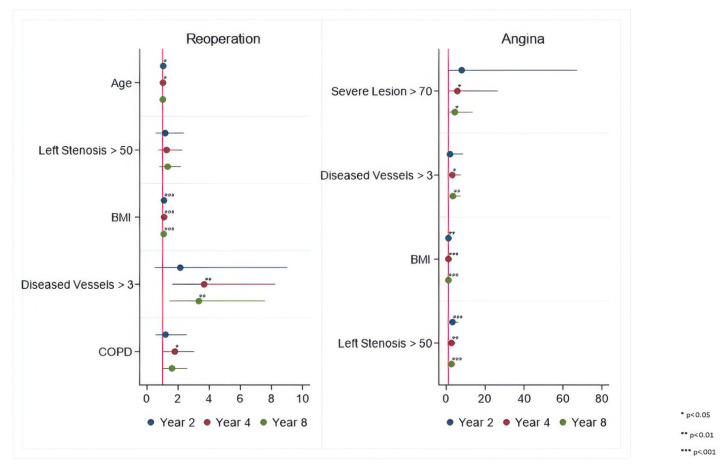
Periprocedural Risk Predictors for Repeat Revascularization and Angina. *—risk factor; **—important risk factor; ***—extremely important risk factor.

To specifically identify risk factors for each of the hazard phases (early, intermediate, and late), the time-varying HR estimates for each outcome were calculated for 2, 4, and 8 years after the surgical procedure, respectively. These are presented in Figure 2, Figure 3 and Figure 4 and Table 4, Table 5, Table 6, Table 7, Table 8 and Table 9.

This analysis revealed that some risk factors such as age > 65 years, COPD, PVD, diabetes, BMI > 30 kg/m^2^, dialysis, low EF < 50%, and >three diseased vessels tended to increase the hazards of all-cause death and MACCE throughout the entire follow-up.

Lack of radial artery graft, smoking, and BMI > 30 kg/m^2^ tend to increase hazards of MI, while left main coronary artery stenosis > 50%, >three diseased vessels, BMI > 30 kg/m^2^, and three disease vessels > 70% have an impact on increasing the hazards of angina throughout the entire follow-up.

Black race and BMI > 30 kg/m^2^ tend to increase the hazards for stroke, while age > 65 years, COPD, >three diseased vessels, BMI > 30 kg/m^2^, and left main coronary artery stenosis > 50% tend to increase hazards for repeat revascularization throughout the entire follow-up.

Thirty-day incidence of repeat revascularization with PCI on surgical target vessels was 0.9% (eight patients), with seven (0.8%) patients being treated for LITA-LAD anastomosis stenosis and one (0.1%) being treated for radial artery graft to diagonal coronary vessel anastomosis stenosis. No patient had repeated surgical revascularization at 30-da follow-up (Table 9). In addition, one stent was used in seven (0.8%) patients, while two stents were used in one (0.1%) patient.

At the 15-year follow-up, 139 patients had repeat revascularization with PCI. Incidence of repeat PCI on LITA-LAD surgical target was 14.4% (20 patients); PCI of the occluded stent in LAD was 2.2% (3 patients), and PCI on LAD for new disease/stenosis was 7.2% (18 patients). In addition, 26 (18.7%) patients had PCI on RCA for in-stent occlusion, while 51 (36.7%) patients had PCI on RCA for new disease/stenosis. The diagonal coronary artery was treated in four (2.9%) patients who had a surgical bypass on the vessel, while one (0.7%) patient had PCI for in-stent stenosis, and six (4.3%) patients had PCI for new disease/stenosis. Finally, 1 (0.7%) patient had PCI in OM/RAMIS, which was a previous surgical target; 19 (13.7%) patients had PCI for in-stent stenosis, and 53 (38.1%) patients had PCI for new disease/stenosis.
**Repeat Intervention at 15-Year Follow-Up****Patient** ***n* = 139**Repeat Intervention with PCI *n* (%)139 (100%)Repeat Surgical Interventions *n* (%)0Patients with at least 1 new vessel disease102 (73.4%)Repeat PCI on LITA-LAD surgical Target *n* (%)20 (14.4%)Repeat PCI on LAD occluded in-stent *n* (%)3 (2.2%)New PCI on new LAD for new disease/stenosis *n* (%)18 (7.2%)Repeat PCI on RCA for in-stent occlusion *n* (%)26 (18.7%)New PCI on RCA for new disease/stenosis *n* (%)51 (36.7%)Repeat PCI on previous Diagonal surgical target *n* (%)4 (2.9%)Repeat PCI on Diagonal for in-stent stenosis *n* (%)1 (0.7%)New PCI on Diagonal for new disease/stenosis *n* (%)6 (4.3%)Repeat PCI on RAMIS/OM previous surgical Target *n* (%)1 (0.7%)Repeat PCI on RAMIS/OM for in-stent stenosis *n* (%)19 (13.7%)New PCI on RAMIS/OM new disease/stenosis *n* (%)53 (38.1%)>PCI—percutaneous coronary interventions; LITA—left internal thoracic artery; LAD—left anterior descending; OM—obtuse marginal; RCA—right coronary artery.

## 4. Discussion

Our clinical study provided several novel insights into the HCR population during a 15-year follow-up:

- More than three-vessel diseases increased the hazard ratio for all-cause mortality, MACCE, repeat revascularization, and angina;
- Severe left main artery stenosis increased the hazard ratio for repeat revascularization and angina after HCR;
- Diabetes had the second highest hazard ratio for all-cause death after HCR and conferred an increased risk throughout the entire follow-up period;
- BMI > 30 kg/m^2^ and age > 65 years increased the hazard for most of the outcomes;
- The mean age was 71.1 years, and more than 8% of the patients underwent more than one graft;
- Intraoperatively, a total of 3.5% of patients received blood transfusion products;
- More than 90% of the patients had a fast-track recovery and were extubated in the OR;
- Total ICU and hospital length of stay were low;
- Postoperatively, only 16% of patients received blood transfusion products;
- Reoperation for bleeding was 0.9%;
- The incidence of stroke was 0.5%.

Firstly, new risk factors are associated with an increased HR for all-cause mortality, MACCE, MI, stroke, repeat revascularization, and angina. Secondly, more than three-vessel disease, diabetes, black race, and obese patients tend to increase the hazard ratio for long-term prognosis, which remains high at 2-, 4-, and 8-year follow-up. Time-varying analysis provides a deeper understanding of the timing of the highest impact of risk factors associated with all-cause death and MACCE in this population. Based on our findings, we speculate that modification and optimization of modifiable risk factors, including diabetes, obesity, and COPD, can improve long-term prognosis [8,9,10].

An analysis conducted by the National Cardiac Surgery Database of the Society of Thoracic Surgeons, using data from CABG operations in over 300,000 patients, showed that obesity was still an independent risk factor associated with an increased time in the operating room and in-hospital mortality rate in patients undergoing CABG [11]. The Reduction in Atherothrombosis for Continued Health (REACH) Registry [12] reported that secondary prevention after CABG may have to be focused on more detailed and exhausting risk factor modifications to bring patients to target goals specified by clinical guidelines [13,14] and reduce the existing variability in risk factor control. In this context, our study reported that obesity is a risk factor in patients with CAD undergoing HCR, and this risk persists at follow-up for the onset of new angina, MI, and the need for repeat revascularization.

Clinical studies have shown that in patients undergoing revascularization, atherosclerosis in grafts and progression of CAD in vessels without grafts are both very common [15,16,17,18]. Of the grafts that were patent at 1 year, about one in three were occluded after 10 years, and an additional one in three were narrowed; in addition, new lesions developed, or old ones progressed in under half the vessels without grafts. In our study, we found that patients with severe three-vessel disease (>70%) and LM coronary artery stenosis > 50% conferred an increased hazard ratio for angina, repeat revascularization, and MI. An internal institutional review of the data evidenced that repeat revascularization due to new disease/non-target vessel disease was around 60%, while the rest was target vessel disease. In this context, Campeau and colleagues’ [19] outcomes suggest that the progression of stenosis in these patients, both in the grafts and native arteries, is an ongoing process.

In our study, we found that patients with more than three vessels conferred an increased hazard ratio for long-term prognosis. In this context, clinical outcomes from the SYNTAX II study evidenced that in patients with three-vessel disease, there was no substantial statistical difference in MACCE between SYNTAX II PCI and matched SYNTAX I CABG patients at 5-year follow-up [20]. The situation becomes even more confused with the latest outcomes from the ISCHEMIA trial [21], which found no difference between an initial invasive strategy compared with an initial conservative strategy, reduced the risk of ischemic cardiovascular events or death from any cause over a median of 3.2 years, even in patients with two- or more vessel disease. Our manuscript suggests that patients with more than two-vessel disease have a higher hazard ratio that impacts long-term outcomes.

Hybrid coronary revascularization is still an evolving field, and more data on postoperative coronary angiography are necessary to further increase the granularity of clinical outcomes. Therefore, a clinical trial designed to assess the impact of risk factors in patients undergoing HCR is crucial in validating the outcomes of this study.

Our clinical study emulates similar risk factors associated with patients with high BMI. In this context [22,23], we believe that less aggressive medical therapy of dyslipidemia with statins may have had an impact on clinical outcomes in patients with high BMI. However, our database did not capture the cholesterol levels in our patients. In addition, our group has previously supported the idea of the impact of race on clinical outcomes in patients undergoing CABG [24,25]. Specifically, we have found that African Americans are at higher risk of complications. The reasons behind that are probably related to the lack of support and education. Therefore, we need to make a call to improve clinical outcomes in this population.

Compared to a multicenter clinical study led by New York State comparing traditional vs. HCR, at a 6-year follow-up, our study showed a hybrid survival rate of 86% (New York State HCR was 80.9%, and traditional CABG was 85.8%) [5]. In addition, repeat intervention in our study was 78% (New York State HCR was 76.6%, and CABG was 88.2%). However, there is a major difference between the mean ages of our study vs. the New York State study, with our patients being 6 years older (our study mean age = 71 years vs. New York State mean age = 65 years for both HCR and traditional CABG). These comparable clinical outcomes between traditional CABG and hybrid PCI have also been found to be significant in a recent clinical review [26].

Postoperatively, goals aim to reduce the impact of risk factors for heart disease. Those include critical strategies to help patients and their families stop smoking, optimal management of the heart rate and blood pressure through B-blockers and ACE inhibitors, improved cholesterol levels through statin therapy and diet, and aggressive control of blood glucose levels in patients with diabetes. In addition, regular exercise and stress management techniques can have a positive impact on patients’ postoperative long-term survival and quality of life. Our group has also previously found that adherence to medical therapy and regular follow-ups had a significant impact on long-term survival and the presence of new symptoms at follow-up [21].

Our group has previously found that morbidly obese patients undergoing CABG had poorer outcomes compared to non-obese patients. We also found that associated risk factors in obese patients undergoing CABG included diabetes, a high Society of Thoracic Surgeons score, chronic dialysis, hypertension, COPD, peripheral vascular disease, EF < 50%, and chronic atrial fibrillation [27]. With respect to HCR, most clinical and functional status results followed the same pattern: the outcomes of HCR were superior to the CORONARY clinical trial and standalone PCI. In addition to the established advantages of HCR over CABG (such as instant confirmation of instant LIMA–LAD graft patency, avoidance of aortic manipulation, standardized dual antiplatelet therapy, the satisfactory long-term patency rate of LIMA–LAD over PCI of LAD, and the superiority of contemporary drug-eluting stents over saphenous vein grafts in terms of long-term patency), we suppose that one of the crucial advantages of HCR lies in the collaboration of the multidisciplinary HCR team, and the individual patient can genuinely benefit from such a decision-making strategy.

## 5. Limitations

This is a retrospective clinical study and is subject to statistical limitations related to a non-randomized study, which may include biases with respect to the type of patients undergoing HCR CABG (mostly due to the lack of clinical guidelines). Other limitations include the lack of a SYNTAX score, the number of implanted stents, postoperative coronary angiography outcomes, and medical therapy. The analysis from this study generates a hypothesis suggesting what factors may have the most value in future research.

## 6. Conclusions

This study shows that patients undergoing MIDCAB HCR may experience all-cause death, stroke, angina, MI, and the need for repeat revascularization over time. Risk factors associated with long-term prognosis include diabetes, COPD, and obesity. Therefore, preoperative medical optimization of modifiable periprocedural risk factors can improve long-term prognosis.

## Figures and Tables

**Table 1:** Preoperative and Intraoperative Characteristics.

Variables	Patients *n* = 875
Female sex *n* (%)	192 (21.9%)
Black race *n* (%)	79 (9.03%)
Mean Age Years (IQR/SD)	71.1 (±11.1)
Age > 75 years *n* (%)	315 (36.0%)
BMI > 30 kg/m^2^ *n* (%)	329 (37.6%)
STS-PROM score % *n* (%)	
Low (<4) *n* (%)	762 (87.1%)
Intermediate (4–8) *n* (%)	73 (8.74%)
High (>8) *n* (%)	40 (4.99%)
eGFR < 90 mL/min/1.73 m^2^ *n* (%)	717 (81.9%)
Preoperative dialysis *n* (%)	26 (2.97%)
Diabetes mellitus *n* (%)	359 (41.0%)
Smoking *n* (%)	420 (48.0%)
COPD *n* (%)	116 (13.3%)
Hypertension *n* (%)	745 (85.1%)
Dyslipidemia *n* (%)	730 (83.4%)
Peripheral vascular disease *n* (%)	116 (13.3%)
Hepatic Failure *n* (%)	11 (1.26%)
Prior PCI *n* (%)	495 (56.6%)
Prior MI *n* (%)	492 (56.2%)
Atrial fibrillation	124 (14.2%)
EF < 50% *n* (%)	201 (23.0%)
Number of diseased vessels > 3 *n* (%)	538 (61.5%)
LM disease *n* (%)	137 (15.7%)
Proximal LAD stenosis > 70% *n* (%)	824 (94.2%)
SVG *n* (%)	11 (1.26%)
Radial artery *n* (%)	46 (5.26%)
Number of grafts > 1 *n* (%)	70 (8.00%)
Stents during planned PCI > 1 *n* (%)	374 (43.7%)
Multiarterial revascularization *n* (%)	60 (6.86%)
Urgent surgery *n* (%)	321 (36.7%)

COPD, chronic obstructive pulmonary disease; eGFR, estimated glomerular filtration rate; LAD, left anterior descending coronary artery; LM, left main coronary artery; MI, myocardial infarction; PCI, percutaneous coronary intervention; STS, Society of Thoracic Surgeons; SVG, saphenous vein graft.

**Table 2:** In-Hospital Outcomes.

In-Hospital Outcomes	Patients *n* = 875
Intra-operative Outcomes	
Time in OR (Hours) (Median/IQR)	5.6 (5.1–6.4)
Blood Transfusion *n* (%)	31 (3.5%)
RBC Units *n* (%)	30 (3.5%)
Cryoprecipitate Units *n* (%)	2 (0.2%)
Platelet Units *n* (%)	5 (0.6%)
FFP Units *n* (%)	0 (0)
Extubated in OR *n* (%)	788 (90.1%)
Postoperative Outcomes	
Total ICU (Hours) (Median/IQR)	29.7 (24.6–54.7)
Total LOS (Days) (Median/IQR)	4 (3–6)
Blood Transfusion *n* (%)	141 (16.1%)
RBC Units *n* (%)	138 (15.8%)
Cryoprecipitate Units *n* (%)	22 (2.5%)
Platelet Units *n* (%)	22 (2.5%)
FFP Units *n* (%)	11 (1.2%)
Stroke *n* (%)	4 (0.5%)
Superficial Infection *n* (%)	0 (0)
Reoperation for Bleeding *n* (%)	8 (0.9%)
Prolonged Ventilation *n* (%)	9 (1.0%)
Renal Failure *n* (%)	4 (0.5%)
Dialysis *n* (%)	0 (0)
New Atrial Fibrillation *n* (%)	144 (16.4%)
30 Day Readmission *n* (%)	87 (10.0%)

OR—operating room; RBC—red blood cells; FFP—fresh frozen plasma; ICU—intensive care unit; LOS—length of stay.

**Table 3:** Overall Analysis of Perioperative Impact of Risk Predictors on Long-term Prognosis.

Variables	All Cause-Mortality HR (95% CI)	MACCE HR (95% CI)	MI HR (95% CI)	Stroke HR (95% CI)	Repeat Revascularization HR (95% CI)	Angina HR (95% CI)
Age Years	1 (1.07, 1.1)	1.04 (1.02, 1.1)	---	---	---	---
BMI kg/m^2^	1.1 (1.02, 1.1)	1.1 (1.04, 1.1)	1.1 (1.03, 1.1)	0.9 (0.8, 0.99)	1.1 (1.3, 3.8)	1.04 (1.02, 1.1)
COPD	2.5 (1.5, 4.0)	2.2 (1.5, 3.1)	---	---	---	---
PVD	1.6 (1.02, 2.7)	1.5 (1.03, 2.1)	---	---	---	---
Diabetes	1.6 (1.0, 2.5)	---	---	---	---	---
Preoperative EF < 50%	1.9 (1.2, 3.0)	---	---	---	---	---
Dialysis	4.8 (2.2, 10.5)	2.6 (1.3, 5.1)	---	---	---	---
Diseased Vessels > 3	3.0 (1.1, 8.5)	2.3 (1.1, 5.0)	---	---	3.0 (1.3, 7.0)	3.2 (1.5, 7.0)
Smoking	---	---	2.5 (1.3, 5.1)	---	---	---
Lack of Radial Artery Graft	---	---	4.8 (1.9, 11.9)	---	---	---
Black Race	---	---	---	3.9 (1.1, 13.9)	---	---
Severe Lesion > 70%	---	---	---	---	2.4 (1.1, 5.5)	---
Left Stenosis > 50%	---	---	---	---	2.2 (1.3, 3.8)	3.4 (1.3, 8.6)

BMI—body mass index; COPD—chronic obstructive pulmonary disease; PVD—peripheral vascular disease; EF—ejection fraction.

**Table 4:** Time-Varying Analysis of Perioperative Risk Factors Associated with MACCE.

Variables	MACCE HR (95% CI)		
Follow-Up Years	2 Years	4 Years	8 Years
Age > 65 Years	1.03 (1.0, 1.1)	1.03 (1.0, 1.1)	1.03 (1.0, 1.1)
BMI > 30 kg/m^2^	1.1 (1.01, 1.1)	1.1 (1.01, 1.1)	1.1 (1.04, 1.1)
COPD	2.2 (1.3, 3.7)	2.5 (1.7, 3.7)	2.2 (1.6, 3.2)
PVD	1.4 (0.8, 2.5)	1.5 (0.9, 2.3)	1.6 (1.1, 2.3)
Dialysis	1.1 (0.3, 3.4)	2.2 (1.0, 4.6)	2.5 (1.3, 5.0)
Diseased Vessels > 3	1.4 (0.3, 5.7)	2.6 (1.1, 5.8)	2.3 (1.1, 4.9)

BMI—body mass index; COPD—chronic obstructive pulmonary disease; PVD—peripheral vascular disease.

**Table 5:** Time-Varying Analysis of Perioperative Risk Factors Associated with MI.

Variables	Stroke HR (95% CI)		
Follow-Up Years	2 Years	4 Years	8 Years
Black Race	1.1 (1.03, 1.1)	1.1 (1.02, 1.1)	1.1 (1.05, 1.1)
BMI > 30 kg/m^2^	0.99 (0.92, 1.1)	1.04 (0.9, 1.1)	1.1 (1.0, 1.1)

BMI—body mass index.

**Table 6:** Time-Varying Analysis of Perioperative Risk Factors Associated with Stroke.

Variables	MI HR (95% CI)		
Follow-Up Years	2 Years	4 Years	8 Years
BMI > 30 kg/m^2^	1.1 (0.9, 1.2)	1.1 (1.1, 1.2)	1.1 (1.03, 1.2)
Smoking	2.7 (0.9, 8.0)	2.3 (0.9, 5.5)	2.3 (1.1, 5.0)
Lack of Radial Artery Graft	7.2 (2.0, 25.6)	7.4 (2.5, 22.4)	6.1 (2.4, 15.2)

BMI—body mass index.

**Table 7:** Time-Varying Analysis of Perioperative Risk Predictors that Impact Repeat Revascularization.

Variables	Repeat Intervention HR (95% CI)		
Follow-Up Years	2 Years	4 Years	8 Years
Age > 65 Years	1.04 (1.0, 1.1)	1.02 (1.0, 1.04)	1.01 (0.99, 1.0)
BMI > 30 kg/m^2^	1.1 (1.04, 1.1)	1.1 (1.1, 1.1)	1.1 (1.04, 1.1)
COPD	1.2 (0.6, 2.5)	1.8 (1.05, 3.0)	1.6 (0.99, 2.6)
Diseased Vessels > 3	2.1 (0.5, 9.0)	3.7 (1.6, 8.2)	3.3 (1.5, 7.6)
Left Stenosis > 50%	1.2 (0.6, 2.4)	1.3 (0.7, 2.2)	1.3 (0.8, 2.2)

BMI—body mass index; COPD—Chronic Obstructive Pulmonary Disease.

**Table 8:** Time-Varying Analysis of Perioperative Risk Predictors that Impact Angina.

Variables	Angina HR (95% CI)		
Follow-Up Years	2 Years	4 Years	8 Years
BMI > 30 kg/m^2^	1.1 (1.01, 1.1)	1.1 (1.03, 1.1)	1.05 (1.02, 1.1)
Diseased Vessels > 3	1.9 (0.4, 8.5)	3.1 (1.3, 7.4)	3.4 (1.5, 7.3)
Severe Lesion > 70%	7.9 (0.9, 67.2)	5.7 (1.2, 26.3)	4.3 (1.4, 13.5)
Left Main Stenosis > 50%	3.1 (1.6, 6.0)	2.6 (1.5, 4.6)	2.6 (1.5, 4.4)

BMI—body mass index.

**Table 9:** Thirty-day Repeat Revascularization Outcomes.

30-Day Outcomes	Patients *n* = 875
30-day PCI intervention *n* (%)	8 (0.9%)
30-day surgical intervention *n* (%)	0
30-day PCI on surgical target vessels *n* (%)	8 (0.9%)
30-day PCI 1 stent on surgical target vessels *n* (%)	7 (0.8%)
30-day PCI 2 stents on surgical target vessels *n* (%)	1 (0.1%)
30-day LITA-LAD PCI *n* (%)	7 (0.8%)
30-day Radial artery graft on diagonal *n* (%)	1 (0.1%)

PCI—percutaneous coronary intervention; LITA—left internal thoracic artery; LAD—left anterior descending.

## Data Availability

The data that support the findings of this study are available upon reasonable request to Serge Sicouri, pending institutional approval.

## Supplementary Materials

The following supporting information can be downloaded at https://www.mdpi.com/article/10.3390/jcdd12010021/s1, Supplemental Table S1: Heart-team interventional approach decision. Supplemental Document S1: STS definitions. Supplemental Document S2: Surgical technique.
