# Supplementary material for: Risk Factors for Coronary Events After Robotic Hybrid Off-Pump Coronary Revascularization
Source: J Cardiovasc Dev Dis. 2025 Jan 10;12(1):21. doi: 10.3390/jcdd12010021 (PMC11765785; doi:10.3390/jcdd12010021)
Supplement: Supplementary file 1 [file jcdd-12-00021-s001.zip › jcdd-3282686-supplementary.pdf]

Document S1: STS definitions

Myocardial Infarction

Myocardial Infarction (MI): (0-24 hours post-op) Indicate the presence of a peri-operative Myocardial Infarction (MI) (0-24 hours post-op) as documented by the following criteria: • The CK-MB (or CK if MB not available) must be greater than or equal to 5 times the upper limit of normal, with or without new Q waves present in two or more contiguous ECG leads. No symptoms required. • (> 24 hours post-op) Indicate the presence of a perioperative MI (> 24 hours post-op) as documented by at least one of the following criteria: 1. Evolutionary ST- segment elevations 2. Development of new Q-waves in two or more contiguous ECG leads 3. New or presumably new LBBB pattern on the ECG 4. The CK-MB (or CK if MB not available) must be greater than or equal to 3 times the upper limit of normal.

Reoperations (cardiac):

Operative re-intervention was required for bleeding/ tamponade, valvular dysfunction, graft occlusion and or other complications.

Sternum infection:

Indicate whether the patient, within 30 days postoperatively, had a deep sternal infection involving muscle, bone, and/or mediastinum REQUIRING OPERATIVE INTERVENTION. Must have ALL of the following conditions:

1. Wound opened with excision of tissue (I&D) or re-exploration of mediastinum
2. Positive culture
3. Treatment with antibiotics

Renal failure:

Indicate whether the patient had acute or worsening renal failure resulting in one or more of the following:

1. Increase of serum creatinine to > 2.0, and 2x most recent preoperative creatinine level.
2. A new requirement for dialysis postoperatively.

Stroke:

Indicate whether the patient has a postoperative stroke (i.e., any confirmed neurological deficit of abrupt onset caused by a disturbance in cerebral blood supply) that did not resolve within 24 hours.

Diabetes:

A history of diabetes, regardless of duration of disease or need for anti-diabetic agents.

Complications:

Indicate whether a postoperative event occurred during the hospitalization for surgery. This includes the entire postoperative period up to discharge, even if over 30 days.

Obesity:

BMI $\geq$30 kg/m^2

Document S2: Surgical technique

Robotic Procedures

Robotic-assisted CABG includes the robotic harvest of the internal mammary artery (IMA) and its direct anastomosis to the coronary artery with a small anterior thoracotomy (4 cm) at the site of the camera port. The robotic procedures started with the introduction of 3 ports in the midclavicular/anterior axillary line, in 2nd, 4th and 6th intercostal space. A camera and two lateral arms with surgical instruments are introduced and the surgeon sits on the robotic console while a table side assistant positions the robotic surgical instruments (Intuitive da Vinci® Robotic). The 3D view offered by the robotic platform enhances the visualization of the IMA, lowering the risk of vessel injury and enables the surgeon to harvest a longer IMA graft by means of skeletonization. If a second IMA is required, the surgeon can open the right pleura crossing the mediastinum and accessing the RIMA from the left side chest. Our technique is based on "incision-precision" (12) where the camera port is located at the precise site of the LAD landing zone. By extending the camera port size to a 4 cm skin incision, the surgeon can perform an off-pump coronary anastomosis using a composite off-pump retractor (Medtronic Octopus® Nuvo Tissue Stabilizer) and off-pump technique to complete the anastomosis. In case two arterial grafts are utilized one side of the second arterial graft is anastomosed to the left internal thoracic artery (LITA) (in a Y or T shape anastomosis fashion) and the other side to the coronary targets (diagonal branch, intermediate branch, and obtuse marginal branch) either sequential or end-to-end. In case a vein

graft is used, the proximal side is anastomosed to the LITA and the distal side to the other coronary targets.

Radial Graft use

The criteria for using a radial graft included a tight proximal coronary stenosis (90%) and adequate OM size.

Hybrid Coronary Procedure

Hybrid coronary revascularization (HCR) treats multivessel coronary artery disease by combining a minimally invasive surgical approach to the left anterior descending artery with percutaneous coronary intervention for non–left anterior descending diseased coronary arteries.

Patients undergoing HCR had their PCI within 1 week from the surgical procedure.

Supplemental Table S1

**Criteria Description**	**Description**
Patients with isolated proximal LAD disease	Patients in which a stent for LAD is considered not ideal due to young age or the length of stent to be deployed would increase the risk of in-stent restenosis

Patient with CTO of the LAD	This can be associated with a second coronary<br>artery disease lesion suitable for PCI/stent
Patients with isolated LM disease AND no or<br>minimal coronary artery disease in different<br>sites	Patients in which a conventional sternotomy<br>CABG would result in CABG with 2 grafts and in which the circumflex artery would not be bypassed with a second arterial conduit
Patients with severe coronary artery disease<br>and advanced age or comorbidities	Patients have multiple comorbidities which,<br>when combined, increase the risk of perioperative complications and possibly<br>mortality (STS PROM 3-10%). LIMA-to-LAD bypass is feasible and recommended by the Heart Team but complete surgical<br>revascularization with sternotomy and CPB<br>may be at elevated risk. Complete revascularization by PCI may not be feasible<br>and medical management may not be appropriate. The comorbidities may include<br>any in combination but are not limited to<br>cerebrovascular disease, peripheral vascular<br>disease, chronic renal insufficiency, osteoporosis, poorly controlled diabetes,<br>advanced age, chronic anemia, autoimmune<br>disorders, recent orthopedic disorder
Patient awaiting transcatheter valve intervention	In this group, patients were referred and<br>accepted for transcatheter valve intervention but were found to have

	significant coronary artery disease. LIMA-to-LAD bypass is recommended, and feasible but combined valve/CABG is at very elevated risk (STS PROM >8%).
Patients with chronic kidney insufficiency with moderate coronary artery disease and involvement of proximal LAD	In this group of patients, LIMA-to-LAD bypass is strongly recommended by the Heart Team and feasible. Creatinine levels are chronically elevated but stable. There is no evidence of acute renal insufficiency. The STS PROM is moderate (3-8%). The patients are not suitable for either complete revascularization by PCI OR PCI to the LAD or left main coronary arteries.
Special circumstances	In this group, patients are candidate for complete surgical revascularization but have circumstances which lead them to request a minimally invasive. For example, patients refuse CABG with sternotomy despite an appropriate conversation of benefits of CABG over PCI with surgeon and interventional cardiologist
